# A Silk Fibroin/Collagen Nerve Scaffold Seeded with a Co-Culture of Schwann Cells and Adipose-Derived Stem Cells for Sciatic Nerve Regeneration

**DOI:** 10.1371/journal.pone.0147184

**Published:** 2016-01-22

**Authors:** Yunqiang Xu, Zhenhui Zhang, Xuyi Chen, Ruixin Li, Dong Li, Shiqing Feng

**Affiliations:** 1 Department of Orthopedics, Tianjin Medical University General Hospital, Tianjin, China; 2 Department of Neurosurgery, Affiliated Brain Hospital of Armed Logistics, Tianjin, China; 3 Institute of Medical Equipment, Academy of Military and Medical Sciences, Tianjin, China; Second University of Naples, ITALY

## Abstract

As a promising alternative to autologous nerve grafts, tissue-engineered nerve grafts have been extensively studied as a way to bridge peripheral nerve defects and guide nerve regeneration. The main difference between autogenous nerve grafts and tissue-engineered nerve grafts is the regenerative microenvironment formed by the grafts. If an appropriate regenerative microenvironment is provided, the repair of a peripheral nerve is feasible. In this study, to mimic the body’s natural regenerative microenvironment closely, we co-cultured Schwann cells (SCs) and adipose-derived stem cells (ADSCs) as seed cells and introduced them into a silk fibroin (SF)/collagen scaffold to construct a tissue-engineered nerve conduit (TENC). Twelve weeks after the three different grafts (plain SF/collagen scaffold, TENC, and autograft) were transplanted to bridge 1-cm long sciatic nerve defects in rats, a series of electrophysiological examinations and morphological analyses were performed to evaluate the effect of the tissue-engineered nerve grafts on peripheral nerve regeneration. The regenerative outcomes showed that the effect of treatment with TENCs was similar to that with autologous nerve grafts but superior to that with plain SF/collagen scaffolds. Meanwhile, no experimental animals had inflammation around the grafts. Based on this evidence, our findings suggest that the TENC we developed could improve the regenerative microenvironment and accelerate nerve regeneration compared to plain SF/collagen and may serve as a promising strategy for peripheral nerve repair.

## Introduction

Despite great improvements in microsurgical techniques, the repair of a peripheral nerve injury (PNI) remains a challenging clinical problem. A PNI is generally treated with autologous nerve transplantation [1,2]. Axon demyelination and degradation occur in the distal stump of the injured nerve after a PNI and lead to dominant muscle atrophy and loss of function [3,4]. Unfortunately, as the “gold standard” treatment for large nerve defects, autologous nerve transplantation also has some inevitable disadvantages, such as tissue availability, secondary deformities, donor-site morbidity, and potential differences in tissue structure and size [5,6]. To date, various strategies have been used in an attempt to stimulate peripheral axonal growth. Cell transplantation therapy has been regarded an effective method because of the release of various growth factors that promote axonal growth [7,8]. In addition, different types of artificial or biological grafts have been developed and investigated to protect axonal regeneration from the infiltration of fibrous scar tissue and to provide suitable mechanical guidance for regenerating nerve fibers [9,10]. Tissue-engineered nerve grafts are typically constructed by a combination of neural scaffolds and various support cells and/or growth factors.

Silk fibroin (SF) and collagen, as two natural and biodegradable materials, have been widely used in peripheral nerve regeneration for many years [11,12]. Schwann cells (SCs) are the main myelin-forming cells in the peripheral nervous system and play a prominent role in neuron survival and function [13]. Like bone mesenchymal stem cells, adipose-derived stem cells (ADSCs) are adult stem cells that can differentiate into osteoblasts, adipocytes, chondrocytes, myocytes, cardiomyocytes and endothelial cells [14]. In particular, ADSCs can be induced into neurospheres and neuronal-like cells in various neurotrophic media [15,16]. Zurita et al. [17] revealed that ADSCs indirectly co-cultured with SCs could realize neural transdifferentiation. Based on this, we introduced a co-culture system of SCs and ADSCs into an SF/collagen scaffold to construct a tissue-engineered nerve conduit (TENC).

## Material and Methods

### Schwann cells: Isolation, culture, and identification

SCs were isolated from the sciatic nerve of neonatal Sprague-Dawley (SD) rats (1–3 d old) under aseptic conditions and then immersed in Hank’s balanced salt solution. The connective tissue and blood vessels were carefully removed under a microscope. The nerves were cut into 1-mm fragments and dissociated with 0.1% collagenase (Sigma, UK) and 0.25% trypsin (Sigma, UK) for 30 min at 37°C. The solution was neutralized in a medium supplemented with 20% (v/v) fetal bovine serum (FBS, Hyclone, USA) and centrifuged at 1000 × g for 5 min. The cell pellet was resuspended in Dulbecco’s modified Eagle’s medium (DMEM) supplemented with 10% FBS and cultured at 37°C in 5% CO_2_. SCs were identified by morphological observation and immunocytochemical labeling of the S100 protein.

### Adipose-derived stem cells: Isolation, culture and identification

Adipose tissue from the inguinal region of adult SD rats was mechanically dissociated and then digested with collagenase type I (Gibco, Carlsbad, CA, USA). The suspension was centrifuged to separate the floating adipocytes from the stromal vascular fraction. The ADSCs were resuspended and cultured at a density of 1 × 10^5^ cells in DMEM supplemented with 10% FBS (Gibco). Cells that did not adhere after 2 d of culture were removed by medium replacement. Rat ADSCs were passaged three to five times before being used for the experiments. The ADSCs after the third passage were evaluated by analytical flow cytometry.

### Preparation of the SF/collagen scaffold

SF was produced using cocoons following the method described elsewhere [18]. In brief, silkworm silk was boiled at 60°C in a 0.5% Na_2_CO_3_ solution for 30 min to remove sericin; thoroughly washed in hot, double-distillated water several times; and stored at 60°C to dry. Purified SF fibers were first dissolved in CaCl_2_⋅CH_3_CH_2_OH⋅H_2_O solution (molar ratio, 1:2:8) at 80°C for 1 h and then centrifuged at 10,000 rpm for 10 min to obtain a clear liquid. The liquid was dialyzed for approximately 72 h and then concentrated for 7 h in 40% polyethylene glycol to the desired concentration. At that point, the solution was ready for preparing the SF/collagen scaffold. Collagen hydrogels at 2.0 mg/ml were prepared as previously described [19]. An SF/collagen blend was prepared in a quality ratio 4:2 of silk fibroin to collagen, and the blended solution was injected into a casting mold (1.5-mm inner diameter, 15 mm in length) followed by demolding through lyophilization. The SF/collagen scaffolds were sterilized by exposure to 20 kGy Co60 and immersed in sterile saline for 30 min before use.

### Characterization of the SF/collagen scaffold

#### Biocompatibility

Scanning electron microscopy was performed to observe the structure of the SF/collagen scaffold and the cells’ growth. After 7 d of culture, the scaffold with attached cells was washed with 0.01 M PBS (pH 7.4), fixed with 4% glutaraldehyde solution and 1% osmic acid at 4°C for approximately 4 h, dehydrated in graded ethanol solutions, and air dried. Finally, the scaffolds were coated with gold using a JEOL JFC-110E Ion Sputter and observed under a scanning electron microscope (Eindhoven, The Netherlands).

#### Mechanical testing

The mechanical properties of the SF/collagen scaffolds were examined using a universal testing machine (Instron5865), and Young's modulus was calculated. The wall thickness of each SF/collagen scaffold was premeasured. The maximum force for pulling out the thread was measured at a constant crosshead speed of 5 mm/min and recorded as the suture retention strength of the specimen [20]. A stress-stain curve was then plotted.

### Animals and surgical procedure

Thirty male adult SD rats weighing between 220 and 250 g were randomly divided into three groups (n = 10/group): those bridged with plain SF/collagen scaffolds (Scaffold group), those bridged with TENCs (TENC group), and those bridged with autografts (Autograft group). SD rats were provided by the Academy of Military Medical Sciences, and the Tianjin Medical University General Hospital Ethics Committee approved this study. The rats were anaesthetized by intraperitoneally injecting 3% pentobarbital sodium (30 mg/kg body weight). After skin preparation and incision, a section of the left sciatic nerve was removed, leaving a 1-cm long defect following the retraction of the nerve ends. The sciatic nerve defects were then repaired by the three different grafts. The removed nerve segment was re-implanted across the nerve defect in the autograft group. Finally, the experimental rats were sacrificed by injecting 3% sodium pentobarbital (50 mg/kg body weight) 12 weeks after surgery.

### Electrophysiological assessment

Twelve weeks after implantation, electrophysiological examinations were performed for all rats in each group as previously described [21]. The sciatic nerve on the operated side was re-exposed under anesthesia. Electrical stimuli were applied to the proximal end of the nerve trunk, and the compound muscle action potential (CMAP) was recorded on the belly of the gastrocnemius muscle. The CMAP was also measured on the contralateral non-operated side and treated as normal control data. The motor nerve conduction velocity (MCV) was calculated from the CMAP amplitude and the distance between the two stimulation sites.

### Examination of myelin sheath

Regenerated nerves were sectioned, fixed in 6% glutaraldehyde, post-fixed with a 1% OsO_4_ solution (Merck KGaA, Darmstadt, Germany), dehydrated in increasing concentrations of ethanol, and embedded in Epon 812 (Electron Microscopy Sciences) epoxy resin. The tissues were embedded in Epon in an oven at 60°C overnight. The samples were cut into semi-thin (500 nm) and ultra-thin (70 nm) sections. The semi-thin sections were stained with 1% toluidine blue (0.1%, Sigma-Aldrich) and examined under a light microscope (Zeiss Axioskop 2 Plus). The ultra-thin sections were stained with 1% uranyl acetate (TAAB, Berkshire, UK) and 1% lead citrate (Ladd) and observed under a transmission electron microscope (JEM-1230, JEOL, Japan).

### Statistical analysis

The data were expressed as the mean ± SEM. The statistical analysis was performed with one-way ANOVA plus Scheffe’s post hoc test. Significance was determined as P < 0.05.

## Results

### Morphology and identification of SCs

**Fig 1:**
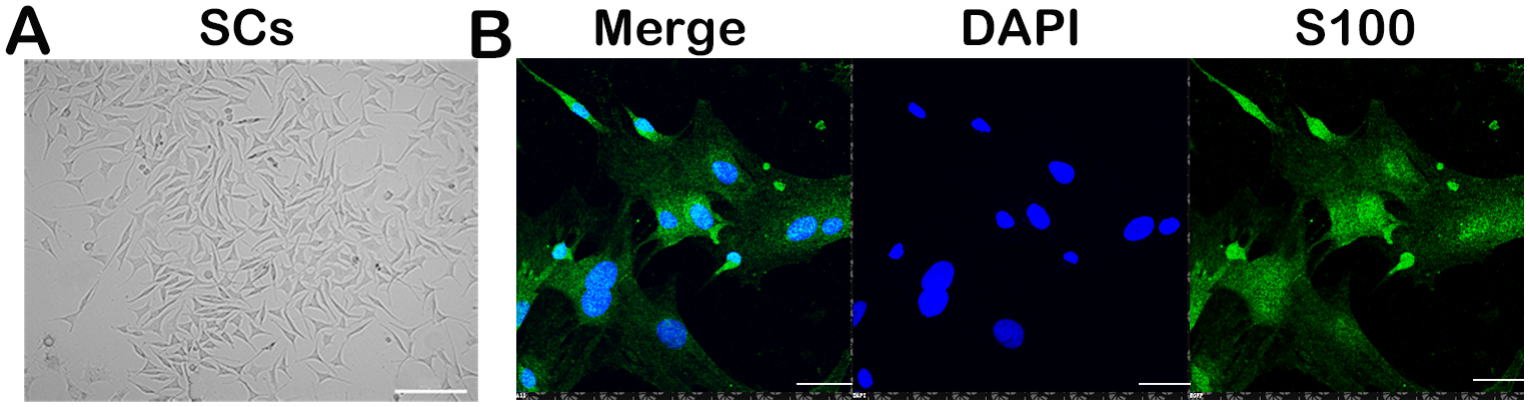
Morphology of Schwann cells. Spindle-elongated morphology of Schwann cells under phase-contrast microscopy (A). An immunocytochemical assessment of Schwann cells shows positivity for the S100 protein (B). Scale bar, 100 (A) and 50 μm (B).

A light microscope was used to identify the SCs. The SCs showed a typical spindle-elongated shape under a phase contrast microscope (Fig 1A). The S-100 protein was regarded as an important and relatively specific marker for SCs [22]. The photographs of the immunocytochemical assessment revealed that the SCs were positive for the S100 protein, and the positive rate was higher than 90% (Fig 1B–1D).

### Morphology and flow cytometric analysis of ADSCs

**Fig 2:**
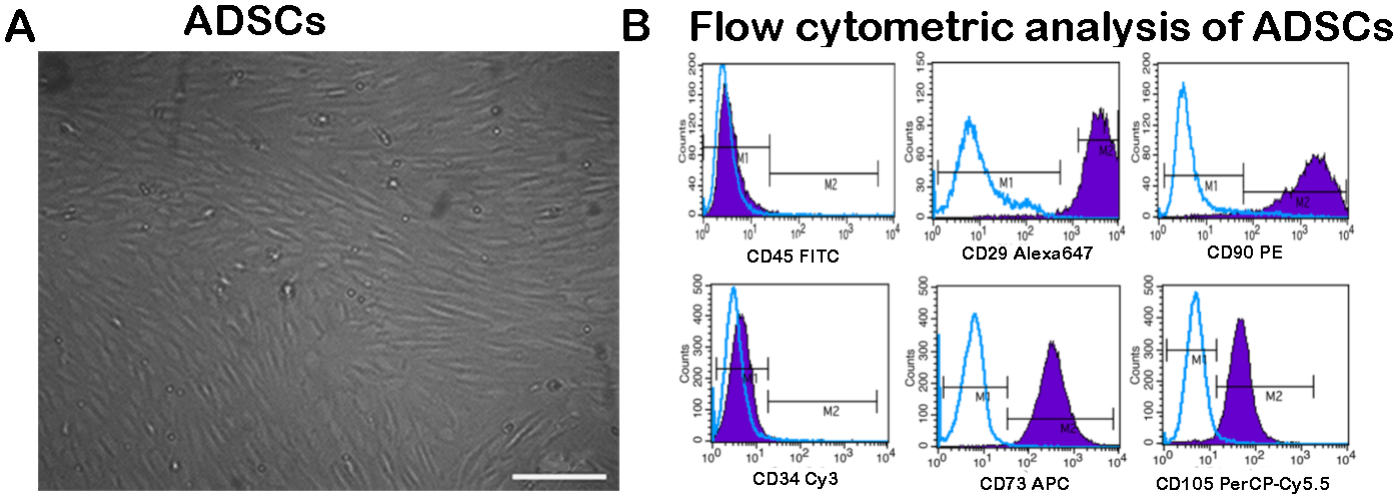
Morphology and flow cytometric analysis of ADSCs. Morphology of ADSCs under phase contrast microscopy (A). Scale bar, 100 μm (A). Flow cytometric analysis of ADSCs (B).

Within 3 to 5 passages after the initial plating of the primary culture, ADSCs became fusiform and exhibited spindle-shaped morphologies (Fig 2A). The ADSCs (3 to 5 passages) were positive for CD29 (99.38%), CD90 (96.21%), CD73 (99.80%), and CD105 (97.97%) but negative for CD34 (0.57%) and CD45 (0.67%) (Fig 2B). The results we obtained are consistent with those of previous studies on ADSCs [23].

### The characterization of the SF/collagen scaffold

#### Scanning electron microscopy

**Fig 3:**
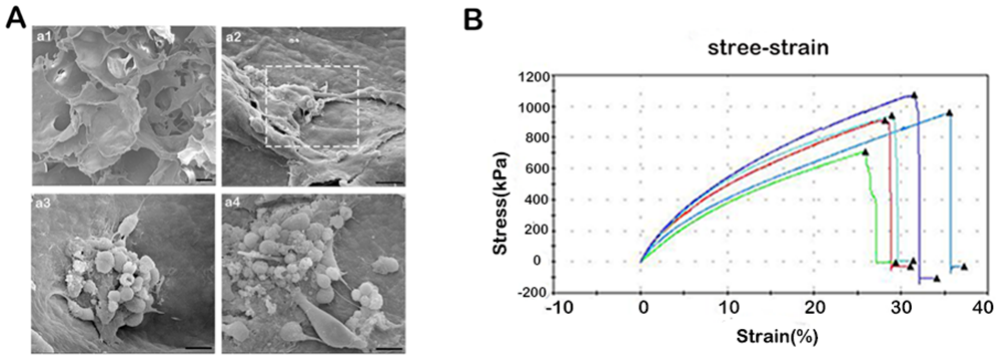
The characteristics of the SF/collagen scaffold. Scanning electron micrographs of a plain SF/collagen scaffold (Fig 3A.a1) and after 7 d of culture of SCs/ADSCs on the SF/collagen scaffold (Fig 3A.a2–3A.a4). Scale bar, 50 (a1), 20 (a2), and 10 μm (a3, a4). The mechanical measurements of the SF/collagen scaffold (Fig 3B).

The plain SF/collagen scaffold shows appropriate pore size and good intercommunicating of holes (Fig 3A.a1). After 7 d of culture, the cells were tightly attached to and partly coiled about the scaffold and exhibited either a spindle or a spherical shape (Fig 3A.a2–3A.a4).

#### Mechanical testing

An ideal neural scaffold should have sufficient mechanical strength to resist in vivo physiological loads after grafting. The results of the mechanical measurement revealed that the maximum and average Young's moduli of the SF/collagen scaffold were 10.81828 ± 0.3 MPa and 8.14551 ± 0.2 MPa, respectively (Fig 3B). The animal experiments confirmed that the mechanical properties ensure that the scaffold can resist muscular contraction and maintain its shape unchanged for a considerable period of time after grafting.

### Electrophysiological analysis

**Fig 4:**
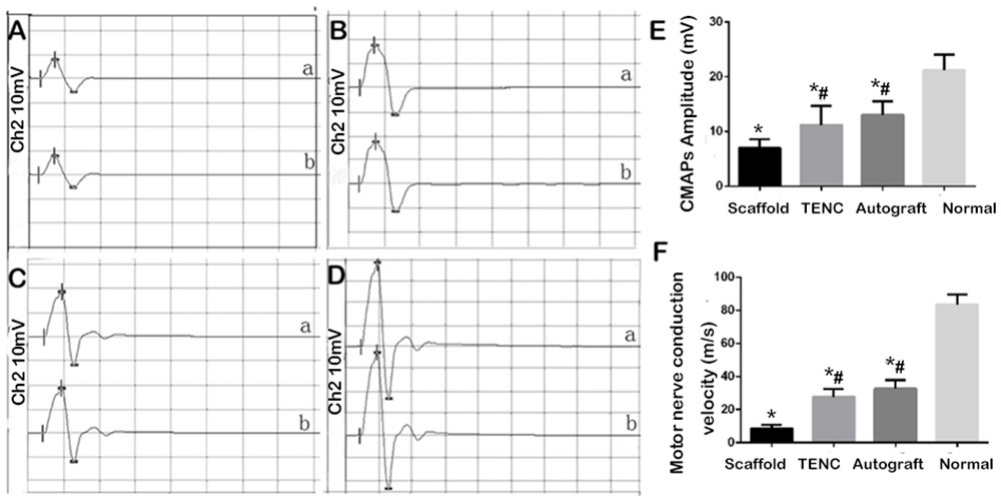
CMAP examinations 12 weeks after surgery. Representative data were recorded on the operated side of animals with plain SF/collagen scaffolds (A), TENCs (B), and autografts (C), and on the contralateral non-operated side (D). Histograms show the CMAP amplitude (E) and the motor nerve conduction velocity (F). Data are expressed as the mean ± SD. #p < 0.05 compared with the Scaffold group, and *p < 0.05 compared with the normal side.

Recordings of CMAP reflected the recovery of the electrophysiological properties of the experimental rats with the three different grafts 12 weeks after surgery. After stimulation at the proximal end of the injured nerve in each group, the CMAP amplitudes and MCVs were significantly lower on the grafted side than on the contralateral non-operated side (Fig 4A–4D). Although no significant differences in CMAP amplitudes and MCVs were detected between the TENC and Autograft groups (P = 0.309 and 0.125, respectively), they were significantly higher in the TENC and Autograft groups than in the Scaffold group (P = 0.026 and 0.0007, respectively) (Fig 4E and 4F). As a significant index for the conduction function of peripheral nerves, the electrophysiological properties could directly reflect the recovery level of the injuried nerve. These results indicate that the TENC-implanted rats achieved similar recovery levels as those in the Autograft group.

### General observations

All experimental animals tolerated the anesthesia and surgery and survived until the end of the observation period. The incisions showed no signs of infection and healed approximately one week after surgery. Three weeks after surgery, all animals in the Scaffold group showed ulcers on the left plantar that had not healed by the end of the experiment, but only three animals in the TENC and Autograft groups showed similar ulcers. The ulcers of the experimental animals were treated with analgesics and bandaged. The animals in the Scaffold group showed more remarkable muscle atrophy on the left hind limbs than those in the TENC and Autograft groups. The composition of the Tianjin Medical University General Hospital Ethics Committee includes the following member:黄东阳: a Doctor of Veterinary Medicine with training and experience in laboratory animal science and medicine; 霍 霞: one practicing scientist experienced in research involving animals; 于晓军: one member from a nonscientific background, drawn from inside or outside the institution; 张 昕: one public member to represent general community interests in the proper care and use of animals;崔玉坤: a legal expert on maintaining animal ethics.

**Fig 5:**
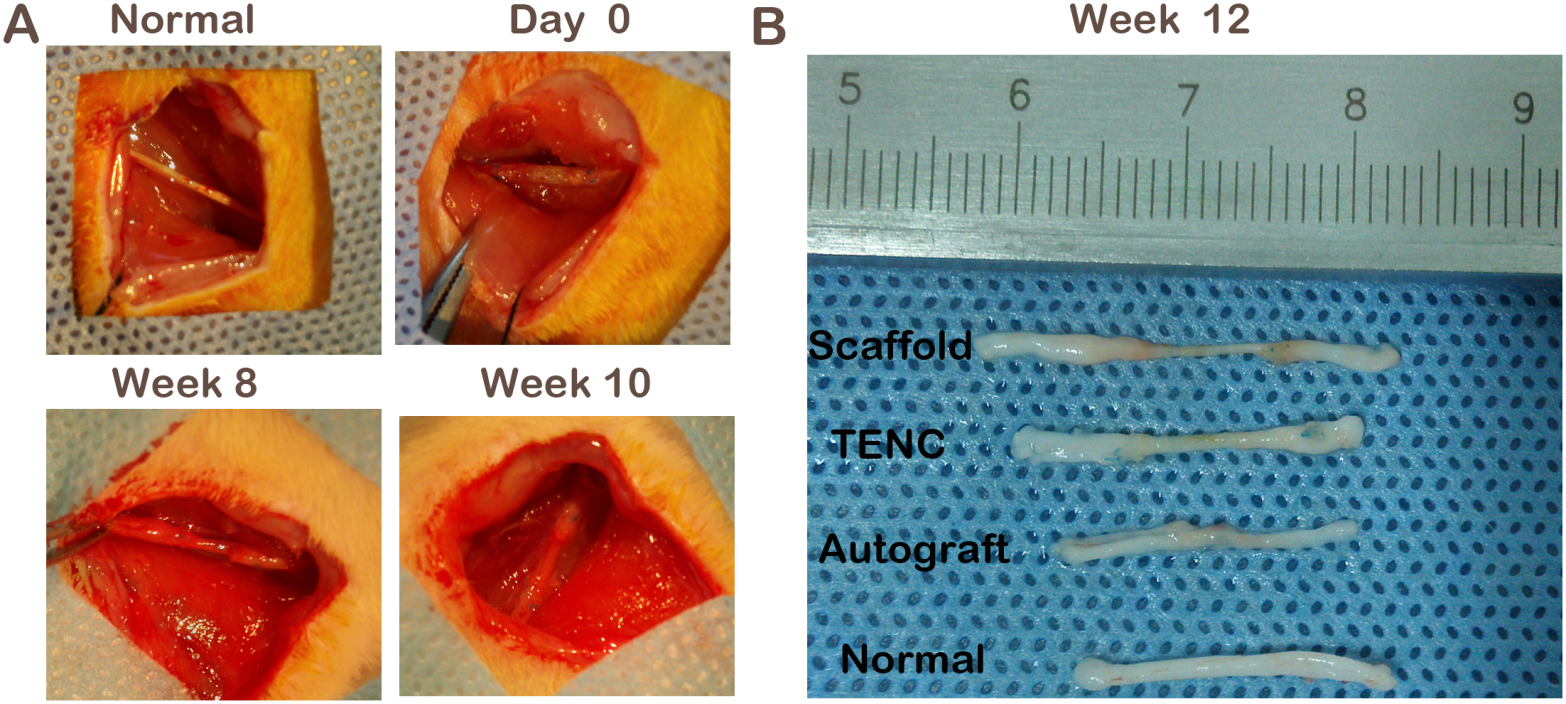
The gross view of sciatic nerve regeneration with different nerve grafts. The change in sciatic nerve regeneration over time (A). The normal sciatic nerve was explored, a 1-cm gap was removed, and the nerve was bridged with different nerve grafts. Examples of regenerated sciatic nerves from plain SF/collagen scaffold, TENC, autograft and control rats (B).

We observed the process of sciatic nerve regeneration over time (Fig 5A). With the SF/collagen scaffolds being absorbed gradually, the new nerves grow across the scaffolds and link up to the operated nerve. In both the Scaffold and TENC groups, most of the grafted rats showed gross nerve regeneration within the scaffolds eight weeks after surgery, and the scaffolds were almost absorbed at the tenth week. The diameter of the regenerated nerves was closely related to the different grafts (Fig 5B). The TENC-grafted rats were similar to those with autografts, with thinner-than-normal nerves, but their nerves were still thicker than those of the plain SF/collagen scaffold-grafted rats.

### Morphological evaluation of regenerated nerves

**Fig 6:**
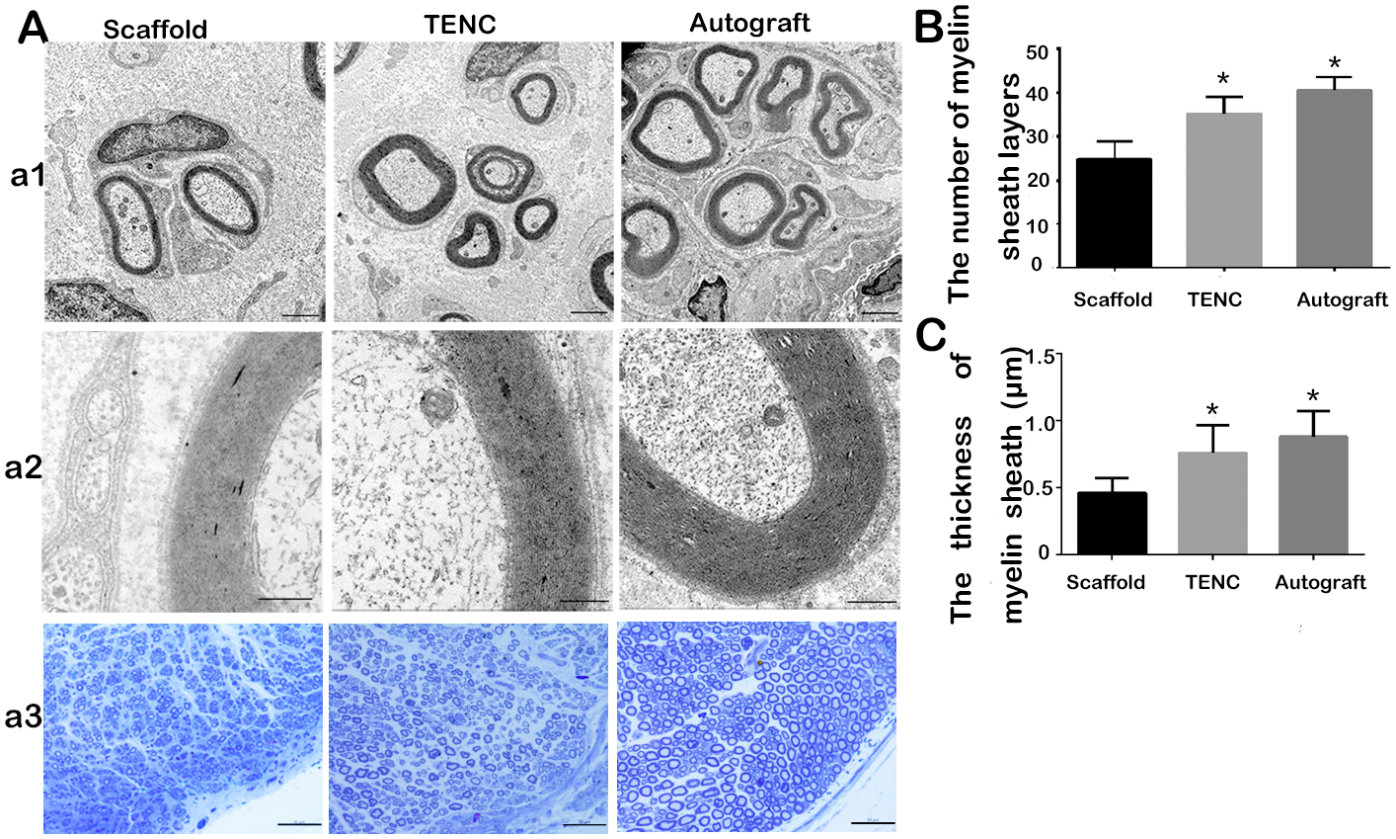
Morphology of the regenerated myelinated nerve fibers. Transmission electron micrographs of the regenerated sciatic nerve (A: a1, a2). Micrographs of toluidine blue staining for the regenerated sciatic nerve under a phase contrast microscope (A: a3). The Scaffold group shows small and poorly developed regenerating clusters composed of thin, dispersed myelinated nerve fibers or non-myelinated nerve fibers. For the TENC group, the regenerated myelinated fibers dispersed densely in clusters and were surrounded by a clear, electron-dense myelin sheath and perfect basal membrane of Schwann cells. Scale bar, 2 (a1), 0.5 (a2), and 20 μm (a3). Histograms showing the number of myelin sheath layers (B) and the thickness of the myelin sheaths (C). Data are expressed as the mean ± SD. *p < 0.05 compared with the Scaffold group.

The semi-thin and ultra-thin sections were observed under a light microscope and transmission electron microscope, respectively. In the Scaffold group, a smaller number of regenerated myelinated nerve fibers were detected compared with the other groups. Transmission electron microscopy and toluidine blue staining indicated that not only were the regenerated myelinated fibers dispersed in clusters, but also the regenerated myelinated axon was surrounded by a clear and electron-dense myelin sheath, although the myelin sheath was thinner than that of a normal nerve (Fig 6A). The myelin lamellar structures provided further evidence for this conclusion. By comparing the morphometric data noted above, we found that neither the number of myelin sheath layers nor the thickness of the myelin sheath showed significant difference between the TENC and Autograft groups (P = 0.091 and 0.302, respectively), but both were significantly higher than in the Scaffold group (P = 0.00017 and 0.00012, respectively) (Fig 6B and 6C).

## Discussion

Tissue-engineering technology is receiving more and more attention in the treatment of PNI. Tissue-engineered nerve grafts may help establish a favorable regenerative microenvironment for guiding and protecting axonal growth and overcome functional donor-site defects caused by autologous nerve transplantation.

In recent years, various synthetic or natural biopolymers (e.g., polylactic acid, polyglycolic acid, alginate and chitosan) and their composites or derivatives have been used to construct nerve scaffolds, but each has its own limitations [24]. Collagen is a natural extracellular matrix component that can provide a suitable surface for cell adhesion and migration [25]. Several investigators have suggested that as a material for nerve scaffolds, collagen has superior biological properties for peripheral nerve regeneration [26]. In particular, collagen has been extensively studied for the repair of peripheral nerve injuries, which led to its clearance by the FDA for use in humans. However, the clinical applications of collagen are limited by its weak manipulability and poor resistance to mechanical forces [27]. Compared with collagen, silk fibroin (SF) is widely used in the field of tissue engineering because of its excellent mechanical properties and rapid biodegradability, and it supports peripheral nerve regeneration [22]. Based on this evidence, a new composite SF/collagen scaffold that maintains the desired biocompatibility and excellent mechanical properties was designed. The maximum and average Young's moduli of the SF/collagen scaffolds were 10.81828 ± 0.3 MPa and 8.14551 ± 0.2 MPa, respectively. The excellent mechanical properties of the rat sciatic nerve allowed the scaffold to resist muscular contraction and keep its shape after grafting into the rats. The scanning electron microscopy (SEM) of the SF/collagen scaffold showed a highly porous structure. On the other hand, SF/collagen scaffolds were also found to support cellular adhesion and proliferation due to their appropriate pore size, which mimics the structure of the extracellular matrix. It is believed that these conduits, which have suitable porosity and oriented morphology, could support nerve regeneration and prevent the failure of defected nerves.

To some extent, the speed of axonal outgrowth determines the peripheral nerve regeneration [28]. Actually, SCs originate from the neural crest and widespread in the peripheral nervous system (PNS). They can form compact myelin around large diameter axons, which are crucial for maintaining the integrity of axons. And axonal maintenance is depend on bidirectional signaling of axons and SCs [29,30]. Once the nerve is injured, SCs begin to proliferate and provide structural and trophic support for axonal regrowth [13]. More importantly, SCs can secrete a variety of neurotrophic factors, such as FGF, NGF, BDNF and GDNF, which make it possible to induce stem cells to differentiate into neuron-like cells when they are co-cultured with SCs [31]. Liao et al. [32] confirmed the capacity of ADSCs for neural differentiation, and these ADSCs can maintain stable and high gene expression levels of GFAP, nestin, and S100 at 14 d after indirect co-culture with SCs. In our previous study, we also confirmed that ADSCs could realize neural transdifferentiation when co-cultured with SCs. Therefore, the co-culture of SCs and ADSCs can not only provide neurons and mimic the native microenvironment for nerve regeneration but also accelerate axonal growth [33].

As an objective and reliable index for the functional recovery of peripheral nerves, the CMAP data for three different graft groups were significantly lower than the normal CMAP data measured on the contralateral non-operated side. Regardless, no significant differences in the CMAP data were detected between the TENC and Autograft groups. The CMAP data of the TENC and Autograft groups were superior to those of the Scaffold group. This implies that not only had enough nerve fibers regenerated and grown across the nerve defects for the three graft groups but also the TENC and Autograft groups reached similar recovery levels. Although the plain SF/collagen conduit could help nerve regeneration, the co-culture of SCs and ADSCs provides a favorable microenvironment for the myelination and growth of the regenerating axons compared with the plain SF/collagen conduit alone. From the gross observation of the regenerated nerve at 8, 10 and 12 weeks after surgery, we can see the change as the regenerated nerve grew into scaffolds and the scaffolds absorbed gradually, and experimental animals showed inflammation around the scaffolds. Finally, the scaffolds were replaced by new regenerated nerves and linked up the 1-cm long sciatic nerve defect stumps. This is consistent with previous reports [34]. Surprisingly, the diameters of the regenerated nerves showed rats with TENCs were similar to those with autografts. This indicates that TENC was more effective in supporting axonal outgrowth than the plain SF/collagen scaffold at the early regenerative stage. The comparison in the images of semi-thin and ultra-thin sections among the 3 grafted groups provided further evidence that the regenerated nerves in the TENC group was closer to those in the Autograft group than that those in the Scaffold group. This might be attributed to the fact that the combination of an SF/collagen scaffold and the co-culture of SCs and ADSCs can mimic the regenerated microenvironment for nerves and accelerate axonal growth.

## Conclusions

We developed a new tissue-engineered nerve conduit (TENC) by combining an SF/collagen nerve scaffold and co-cultured Schwann cells and adipose-derived stem cells to bridge a 1-cm gap in the rat sciatic nerve transection model. Although the desired nerve regeneration was achieved with TENC, the recovery of function (e.g., muscle atrophy, tactile allodynia and thermal hyperalgesia) was still unsatisfactory. We will further explore the functional recovery after PNI in our next study.
